# Agreement of general practitioners with the guideline-based stepped-care strategy for patients with osteoarthritis of the hip or knee: a cross-sectional study

**DOI:** 10.1186/1471-2296-14-33

**Published:** 2013-03-11

**Authors:** Agnes J Smink, Sita MA Bierma-Zeinstra, Joost Dekker, Thea PM Vliet Vlieland, Johannes WJ Bijlsma, Bart A Swierstra, Joke H Kortland, Theo B Voorn, Cornelia HM van den Ende, Henk J Schers

**Affiliations:** 1 Department of Rheumatology, Sint Maartenskliniek, PO box 9011, 6500 GM Nijmegen, The Netherlands; 2 Department of General practice and Department of Orthopaedics, Erasmus University Medical Center, Rotterdam, The Netherlands; 3 Department of Rehabilitation Medicine, VU University Medical Center, Amsterdam, The Netherlands; 4 Department of Rheumatology and Department of Orthopaedics, Leiden University Medical Center, Leiden, The Netherlands; 5 Department of Rheumatology and Clinical Immunology, University Medical Center, Utrecht, The Netherlands; 6 Department of Orthopaedics, Sint Maartenskliniek, Nijmegen, The Netherlands; 7 Dutch patient organization for rheumatic diseases, Amersfoort, The Netherlands; 8 Department of Primary and Community Care, Radboud University Nijmegen Medical Center, Nijmegen, The Netherlands

## Abstract

**Background:**

To improve the management of hip or knee osteoarthritis (OA), a multidisciplinary guideline-based stepped-care strategy (SCS) with recommendations regarding the appropriate non-surgical treatment modalities and optimal sequence for care has been developed. Implementation of this SCS in the general practice may be hampered by the negative attitude of general practitioners (GPs) towards the strategy. In order to develop a tailored implementation plan, we assessed the GPs’ views regarding specific recommendations in the SCS and their working procedures with regard to OA.

**Methods:**

A survey was conducted among a random sample of Dutch GPs. Questions included the GP’s demographical characteristics and the practice setting as well as how the management of OA was organized and whether the GPs supported the SCS recommendations. In particular, we assessed GP’s views regarding the effectiveness of 14 recommended and non-recommended treatment modalities. Furthermore, we calculated their agreement with 7 statements based on the SCS recommendations regarding the sequence for care. With a linear regression model, we identified factors that seemed to influence the GPs’ agreement with the SCS recommendations.

**Results:**

Four hundred fifty-six GPs (37%) aged 30–65 years, of whom 278 males (61%), responded. Seven of the 11 recommended modalities (i.e. oral Non-Steroidal Anti-Inflammatory Drugs, physical therapy, glucocorticoid intra-articular injections, education, lifestyle advice, acetaminophen, and tramadol) were considered effective by the majority of the GPs (varying between 95-60%). The mean agreement score, based on a 5-point scale, with the recommendations regarding the sequence for care was 2.8 (SD = 0.5). Ten percent of the variance in GPs’ agreement could be explained by the GPs’ attitudes regarding the effectiveness of the recommended and non-recommended non-surgical treatment modalities and the type of practice.

**Conclusion:**

In general, GPs support the recommendations in the SCS. Therefore, we expect that their attitudes will not impede a successful implementation in general practice. Our results provide several starting points on which to focus implementation activities for specific SCS recommendations; those related to the prescription of pain medication and the use of X-rays. We could not identify factors that contribute substantially to GPs’ attitudes regarding the SCS recommendations regarding the sequence for care.

## Background

Osteoarthritis (OA) of the hip or knee is a common joint disorder causing pain and functioning impairment. The core treatment for OA, a combination of pharmacological and non-pharmacological treatment modalities, is mainly performed in primary care. In the Netherlands, once the diagnosis had been established, it has been found that patients with OA are treated in general practice for approximately 82 months before they are referred to an orthopaedic department [1]. Although several clinical practice guidelines (CPGs) to manage hip or knee OA exist [2-6], diagnostic procedures, referrals, and use of treatment modalities observed in primary care tend to be inadequate [7-9]. In addition, a recent review depicts a notably low pass rate for quality indicators for OA care (interquartile range 29-41%) [10]. These findings support the conclusion that incorporation of CPGs into clinical practice is not that simple.

We have developed a multidisciplinary, guideline-based stepped-care strategy, known as BART, i.e. Beating Osteoarthritis, to improve the management of hip or knee OA [11]. Experts from each discipline involved in OA care, as well as representatives from patient organizations and professional associations, participated in the development. This stepped-care strategy (SCS) provides a framework for various health care providers and patients to manage hip or knee OA (see Table 1). In addition to the current CPGs that give recommendations about the appropriate non-surgical treatment modalities, the SCS focuses also on the optimal order in which to employ them. This sequence is presented in three steps. At each step, recommendations for diagnostic procedures, treatment modalities, and the length of treatment before evaluation are made. Consequently, more advanced options are only recommended if the options listed in previous steps have failed to produce satisfactory results. Despite a possible risk of delay for more advanced modalities, the SCS is a suitable approach for patients with hip or knee OA.

**Table 1:** **Summary of the SCS recommendations in each step **[11]

	**Step 1**	**Step 2**	**Step 3**
Diagnostic procedures and assessment	- Medical history and physical examination	- Radiological assessment^*^	- Consultation specialist
	- Assessment function and activity limitations	- Assessment of pain coping and psychosocial factors	- Adjust goals
	- Setting mutual goals	- Adjust goals	
Treatment modalities	- Education	- Exercise therapy	- Multidisciplinary care
	- Lifestyle advice	- Dietary therapy	- TENS
	- Medication^†^	- Medication ^†^	- Medication^†^
	∙ *Acetaminophen*	∙ *(topical) NSAID’s*	∙ *Intra-articular injections*
	∙ *Glucosaminesulphate*	∙ *Tramadol*	
Evaluation	-After 3 months^‡^	-After 3–6 months^‡^	-Patient sets interval

Abbreviations: SCS = stepped care strategy; NSAIDs = Non-Steroidal Anti-Inflammatory Drugs.

^*^ If there is a discrepancy between medical history and physical examination.

^†^ Consult current guidelines for an adequate dose.

^‡^ Or earlier if the symptoms persist or increase.

To implement this strategy, the views about the SCS recommendations and working procedures concerning OA care of general practitioners (GPs) need to be assessed and the barriers that prevent GPs from using the SCS need to be identified [12-14]. This knowledge can then be used to develop implementation activities tailored for GPs. The importance of such activities, created in response to identified barriers, has been demonstrated in a recent review that reported that tailored activities are more likely to improve professional practice than non-tailored activities [15]. Also, insight into the GPs’ views provides information concerning the strengths and weaknesses of current practice and heightens the perception of the need for change [13].

An approach based on a theoretical model can help to identify these barriers. Barriers are frequently grouped into social factors (including patients’ preferences), the clinicians’ attitudes, the implementation process, accessibility and format of the program, and external barriers of a practical nature [12,16-18]. In this study, we assessed the attitudes towards the SCS recommendations expressed by GPs which could be a potential barrier to successful implementation of this strategy [19]: specifically, their attitudes regarding the effectiveness of recommended non-surgical treatment modalities for hip or knee OA and their degree of agreement with recommendations regarding the sequence for care. Considering the fact that each recommendation can be influenced by different factors [20], we will assess the attitudes towards several specific SCS recommendations.

The aim of this study is to describe the GPs’ attitudes with respect to the two key elements of the SCS: 1) their attitudes regarding the effectiveness of the recommended non-surgical treatment modalities and 2) their agreement with specific recommendations regarding the sequence for care. In particular, those factors, that could influence their agreement with these recommendations, will be identified and will be used to develop tailored made implementation activities for this target group.

## Methods

### General practitioners

To estimate the prevalence of GPs who agree with the SCS recommendations with 95% confidence level and a maximal error margin of 5%, data of 369 GPs was needed for this cross-sectional study. Assuming a response rate of 30%, a random sample of 1230 GPs was drawn from all listed GPs in the Netherlands (about 8.900) by the Netherlands Institute for Health Services Research (NIVEL) in July 2011. The anonymous survey was sent to this sample, followed by a reminder after two weeks to maximize the response.

### Survey

The survey consisted of questions regarding the GP’s characteristics and their practice setting, as well as the organization of OA management and the GP’s attitudes towards the SCS recommendations.

#### GP’s characteristics and their practice setting

The demographic characteristics included age, sex, length of time working (expressed in years), number of working hours (expressed in fulltime equivalents (fte)), and their field of special interest (e.g. in musculoskeletal disorders (yes/no)). Practice characteristics included type of practice (solo, duo, practice group, GP centre, and health centre), location of the practice (urban, suburban, or rural), practice size (expressed in number of registered patients), number of GPs working in practice (expressed in fte), presence of practice staff (e.g. practice nurse and assistant (yes/no)) as well as other health care providers in the practice (e.g. physical therapist, dietician, physiologist, pharmacist, social worker (yes/no)).

#### Organization of OA management

The organization of OA management in general practice was assessed by mapping the involvement of the GP, practice nurse, and practice assistant in the following care tasks: a) providing information, b) providing lifestyle advice, c) distributing patient information material from the Dutch College of General Practitioners (NHG), d) distributing other types of information, e) referral to a dietician, f) referral to a physical or exercise therapist, and g) evaluating results at the follow-up appointment.

In addition, we assessed the type of collaboration the GP had with other health care providers involved in OA care (physical or exercise therapists, dieticians, rheumatologists, and orthopaedic surgeons) by using three items: 1) participation in periodic meetings concerning individual OA patients (yes/no), 2) following protocols or agreements concerning specific working procedures to treat OA patients (yes/no), and 3) the frequency of contact (rarely, yearly, monthly, weekly) concerning individual OA patients.

#### GP’s attitudes towards the SCS recommendations

We studied GPs’ attitudes towards the two key elements of the SCS: their attitude concerning the appropriate treatment and their attitude concerning the optimal sequence. Therefore, we assessed which of the frequently-used treatment modalities were found to be effective in the treatment of hip or knee OA by the GPs. Furthermore, we assessed if GPs agreed with the sequence of care that is presented in the SCS recommendations.

GP’s attitudes regarding the effectiveness of fourteen frequently-used treatment modalities were scored on a 4-point Likert scale (i.e. not effective – effective) or not applicable (“no experience with the modality”). Eleven of these modalities are recommended in the SCS (education, lifestyle advice, acetaminophen, glucosaminesulphate, oral and topical Non-Steroidal Anti-Inflammatory Drugs (NSAIDs), physical therapy, tramadol, transcutaneous electrical nerve stimulation (TENS), hyaluronic acid, and glucocorticoid injections). Three other frequently-used modalities (massage, manual therapy, and other passive physical therapy treatment modalities, such as cold or heat therapy, ultrasound, laser therapy, or electrotherapy) are not recommended in the SCS, i.e. non-recommended modalities.

GP’s attitudes regarding the sequence for care was assessed by a 5-point Likert scale (i.e. strongly disagree, disagree, neutral, agree, and strongly agree) for seven statements. These statements were based on the SCS recommendations and thus concerned the three areas of management: diagnosis (statement 1), treatment (statement 2–6), and evaluation (statement 7). Treatment modalities from all different steps of the SCS were covered: step 1 (statement 3), step 2 (statements 2 and 4), step 3 (statement 5), and “step 4” (statement 6).

### Data analysis

We used descriptive statistics for the GP’s characteristics and their practice setting, the organization of OA management, and the GP’s attitudes towards the SCS recommendations.

In order to examine the collaboration of the GPs with other health care providers, we constructed two variables by combining items. First, we considered collaboration “structural” (yes/no), if the GP reported to have periodic meetings or reported to follow protocols or agreements concerning specific working procedures with any other health care provider. Second, we considered collaboration “ad hoc” (yes/no), if the GP had at least monthly contact with one or more of the other health care providers.

Furthermore, we constructed two indices to examine the GPs’ attitudes regarding the effectiveness of the fourteen frequently-used non-surgical treatment modalities. One index concerned their attitude regarding the effectiveness of the eleven modalities that are recommended in the SCS, while the other index concerned their attitude regarding the effectiveness of the three non-recommended treatment modalities. We calculated an average score for both indices based on the results on the 4-point Likert scales. If more than one-third of the items was missing (i.e. four or more items for the first index and two or more items for the second index), the scores (range of 0–3; in which 0 = “negative attitude” and 3 = “positive attitude” regarding the effectiveness of the corresponding modalities) were treated as being missing. We excluded items that were ‘not applicable’ from the average score.

Finally, we used linear regression models to assess univariable and multivariable associations between the GP’s agreement with recommendations regarding the sequence for care and the characteristics relating to the GPs, the practice, or organization of management. We constructed an overall index for GPs’ agreement with the seven statements by calculating the average score of items (range of 0–4; in which 0 = “no agreement” and 4 = “complete agreement” with the SCS recommendations). For that matter, the scores on the items in which the desired response was “disagree” were reversed. The scores were treated as being missing if more than one-third of the items was missing (i.e. three or more items). The results were expressed in betas with standard error (SE). All variables that showed univariable significance (p < 0.10) were entered simultaneously into a multivariable model. Statistical analyses were executed using STATA 10.0.

## Results

Out of the 1230 approached GPs, 456 GPs (37%) responded to the survey. Differences between the main characteristics of the participating GPs and the total population of Dutch GPs [21] were limited to the number of working hours and location of practice (Table 2).

**Table 2:** Characteristics of the responders (N = 456) and the total population Dutch GPs (N = 8884)

	**Participating GPs**	Total population Dutch GPs^*^
Age (years); mean	49	48
Sex (male); %	61	59
Working hours (fte); %		
- <0.6	14	18
- 0.6-0.8	28	22
- >0.8	58	61
Type of practice; %		
- Solo	20	18
- Duo	28	28
- Group	51	54
Location practice; %		
- Urban	41	48
- Suburban	20	19
- Rural	38	34

Abbreviations: GPs = General practitioners; N = Number of GPs; fte = fulltime equivalents.

^*^ Poll 1 January 2011 [21].

### GP’s characteristics and their practice setting

Table 3 presents a summary of the characteristics of the participating GPs and their practice setting. Most participating GPs (62%) reported to have one or more fields of special interest. Palliative care (27%), diabetes mellitus (18%), asthma/COPD (17%) cardiovascular diseases (16%), and musculoskeletal disorders (15%) were the most frequently reported fields of interest.

**Table 3:** Characteristics responding GPs and their practice setting (N = 456)

		
**Characteristics of the GP**		
Age (years); mean (SD)	49 (9)	
Sex (male); N (%)	278 (61)	
Length of time working (years); median (IQR)	16 (9–25)	
Working hours (fte); N (%)		
- <0,6	63 (14)	
- 0,6-0,8	125 (28)	
- >0,8	263 (58)	
GPwSI in musculoskeletal disorders; N (%)	69 (15)	
**Characteristics of the practice setting**		
Type of practice; N (%)		
- Solo	92 (20)	
- Duo	127 (28)	
- Practice group	64 (14)	
- GP centre	118 (26)	
- Health centre	49 (11)	
Location practice		
- Urban	189 (41)	
- Suburban	92 (20)	
- Rural	175 (38)	
Practice size (registered patients); median (IQR)	4175 (2700–7000)	
Number of GPs working (fte); median (IQR)	2.0 (1.2–3.6)	
Presence of practice staff; N (%)		
- Practice nurse	403 (88)	
- Practice assistant	440 (96)	
Presence of other disciplines in same setting; N (%)		
- Physical therapist	182 (40)	
- Dietician	195 (43)	
- Psychologist	169 (37)	
- Other (e.g. pharmacist, social worker, dentist)	148 (32)	
**Organization of OA management**		
Involvement of other disciplines in OA management; N (%)		
- Practice nurse	78 (17)	
- Practice assistant	82 (18)	
Structural collaboration; N (%)		
- Periodic meetings	98 (22)	
- Following protocols and agreements on working procedures	55 (12)	
Frequency of contact with other disciplines in OA management; N (%)		
- (Almost) never	100 (22)	
- Yearly	119 (27)	
- Monthly	188 (42)	
- Weekly	39 (9)	

Abbreviations: GPs = General practitioners; SD = standard deviation; N = number of GPs; IQR = interquartile range; fte = fulltime equivalents; GPwSI = GP with a special interest; OA = Osteoarthritis.

### Organization of OA management

Of the 403 GPs (88%) who have a practice nurse available, 73 GPs (18%) reported that their practice nurse is involved in the OA management: “providing lifestyle advice” was the most frequently reported performed task. Of the 440 GPs (96%) who have a practice assistant available, 79 GPs (18%) reported that their practice assistant is involved in OA management: their most frequently performed task in OA management was “handing out other kind of information”.

One hundred twenty-two GPs (27%) reported having “structural” collaboration (having periodic meetings or following protocols or agreements concerning specific working procedures) with other health care providers. Two hundred twenty-seven GPs (51%) reported having “ad hoc” (at least monthly) contact with other health care providers. Both, structural and ad hoc collaboration, were generally with a physical therapist. With regard to structural collaboration, 96 (98%) of the GPs who reported having periodic meetings reported those to be with physical therapists, and 52 (95%) of the GPs who reported following protocols or agreements concerning specific working procedures reported those to be with physical therapists. In addition, 214 GPs (95%) of the GPs who reported having ad hoc contact reported that to be with physical therapists. Thirteen (4%), 38 (10%), and 64 (16%) of the GPs reported having ad hoc contact with a dietician, rheumatologist, or orthopaedic surgeon, respectively.

### GPs’ attitudes towards the SCS recommendations

Seven of the recommended modalities (i.e. oral NSAIDs, physical therapy, glucocorticoid intra-articular injections, education, lifestyle advice, acetaminophen, and tramadol) were considered effective by the majority of the GPs (varying between 95-60%) (Table 4). Fewer GPs found the non-recommended modalities to be effective (39-14%).

**Table 4:** GPs’ attitudes regarding the effectiveness of OA treatment modalities

**Treatment modalities**	**Not effective N (%)**	**Effective N (%)**	**No experience N (%)**
**Modalities recommended in the SCS**			
1. Education	45 (10)	397 (89)	5 (1)
2. Lifestyle advice	49 (11)	393 (88)	6 (1)
3. Acetaminophen	78 (17)	371 (83)	0 (0)
4. Glucosamine^*^	343 (76)	75 (17)	32 (7)
5. Oral NSAIDs	24 (5)	428 (95)	0 (0)
6. Topical NSAIDs	237 (53)	111 (25)	102 (23)
7. Tramadol	172 (38)	269 (60)	10 (2)
8. Physical therapy	35 (8)	416 (92)	0 (0)
9. Glucocorticoid intra-articular injections^**†**^	30 (7)	404 (90)	16 (4)
10. Hyaluronic acid intra-articular injections^**†**^	94 (21)	100 (23)	249 (56)
11. TENS^‡^	115 (26)	123 (27)	212 (47)
**Modalities not recommended in the SCS**			
12. Massage	328 (73)	62 (14)	60 (13)
13. Manual therapy	280 (62)	125 (28)	45 (10)
14. Other passive physical therapy modalities^§^	155 (34)	175 (39)	121 (27)

Abbreviations: GPs = General practitioners; OA = Osteoarthritis; N = number of GPs; SCS = Stepped-care strategy; NSAIDs = Non-Steroidal Anti-Inflammatory Drugs; TENS = Transcutaneous Electrical Nerve Stimulation.

^*^ The SCS suggests the possibility of a trial period of 3 months.

^†^ Intra-articular injections can be considered in patients with knee OA.

^‡^ The SCS only suggests TENS if exercise therapy and medication have not resulted in pain reduction.

^§^ Passive physical therapy modalities, like cold or heat therapy, ultrasound, laser or electrotherapy.

The highest level of agreement with SCS recommendations regarding the sequence for care was reported for the statements 2 and 4; 86% of the GPs (strongly) disagreed with the statement *‘NSAIDs should only be prescribed if there is radiological OA’* and 91% of the GPs (strongly) disagreed with the statement *‘Physical therapy should only be prescribed if there is radiological OA’* (Table 5). The highest level of disagreement with the SCS recommendations was reported for the statement 1 and 3: 48% of the GPs (strongly) agreed with statement *‘X-ray is necessary to diagnose OA’* and 21% of the GPs (strongly) agreed with the statement *‘NSAIDs should be the first choice of pain medication in patients with OA’*. The average score (SD) for the seven statements regarding the sequence for care, was 2.8 (0.4) on a 5-point scale.

**Table 5:** GPs’ agreement with SCS recommendations regarding the sequence for care

**Statements**	**Strongly disagree**	**Disagree**	**Neutral**	**Agree**	**Strongly agree**	Desirable response^*^
	**N (%)**	**N (%)**	**N (%)**	**N (%)**	**N (%)**	
1. X-ray is necessary to diagnose OA	14 (3)	119 (26)	100 (22)	177 (39)	42 (9)	Disagree
2. NSAIDs should only be prescribed if there is radiological OA	167 (37)	220 (49)	45 (10)	13 (3)	7 (2)	Disagree
3. NSAIDs should be the first choice of pain medication in patients with OA	137 (30)	141 (31)	77 (17)	74 (16)	22 (5)	Disagree
4. Physical therapy should only be prescribed if there is radiological OA	202 (45)	206 (46)	32 (7)	9 (2)	3 (1)	Disagree
5. Intra-articular injections should only be prescribed if physical therapy and painkiller are insufficient	15 (3)	65 (14)	77 (17)	200 (44)	94 (21)	Agree
6. Surgical treatment modalities should only be considered if physical therapy and painkiller are insufficient	6 (1)	25 (6)	36 (8)	207 (46)	178 (39)	Agree
7. OA patients should be stimulated by their GP to evaluate and monitor their treatment	5 (1)	27 (6)	88 (20)	195 (43)	135 (30)	Agree

Abbreviations: GPs = General practitioners; SCS = Stepped-care strategy; OA = Osteoarthritis; NSAIDs = Non-Steroidal Anti-Inflammatory Drugs.

^*^ According to the SCS.

### Determinants for a GP’s agreement with the SCS

An univariable association was found between GP’s agreement with the SCS recommendations regarding the sequence for care and four of the characteristics relating to the GP. These were the attitudes towards the effectiveness of recommended and non-recommended treatment modalities, the type of practice, and whether the GP had structural collaboration with other health care providers (Table 6). The multivariable analysis revealed that a positive attitude towards the effectiveness of recommended modalities, a negative attitude regarding the effectiveness of non-recommended modalities, and working in a duo or group practice were significantly associated with a higher agreement with SCS recommendations regarding the sequence for care. Together these three variables explained 9.5% of the variance in this model.

**Table 6:** Uni- and multivariable associations between potential barriers of GPs agreement with the SCS recommendations about the sequence for care

	**Univariable analysis**			**Multivariable analysis**		
	**Beta**	**(SE)**	**p-value**	**Beta**	**(SE)**	**p-value**
**GP’s characteristics**						
Length of time working, years; median (range)	−0.00	(0.00)	0.87			
GPwSI in musculoskeletal disorders; N (% yes)	0.06	(0.06)	0.29			
*Effectiveness recommended modalities (range 0–3); mean (SD)*	*0.18*	*(0.07)*	*0.01*	*0.23*	*(0.07)*	*0.00*
*Effectiveness non-recommended modalities (range 0–3); mean (SD)*	*−0.14*	*(0.04)*	*0.00*	*−0.16*	*(0.04)*	*0.00*
**Practice setting**						
Number of GPs working; median (range)	0.00	(0.01)	0.90			
Number of registered patients (per 1000); median (range)	0.01	(0.01)	0.35			
*Solo practice; N (% yes)*	*−0.15*	*(0.05)*	*0.00*	*−0.15*	*(0.05)*	*0.01*
Availability practice nurse; N (% yes)	0.06	(0.07)	0.38			
Number of other disciplines available; median (range)	0.01	(0.01)	0.52			
**Organization of OA management**						
Structural collaboration; N (% yes)	0.08	(0.05)	0.08	0.89	(0.05)	0.08
Ad hoc collaboration; N (% yes)	−0.00	(0.04)	0.97			

Abbreviations: GPs = General practitioners; SCS = Stepped-care strategy; GPwSI = GP with a special interest; N = number of GPs; SD = standard deviation; OA = Osteoarthritis.

Note: The italic numbers are statistically significant.

## Discussion

In general, the GPs’ attitudes were in concordance with SCS recommendations regarding the non-surgical treatment modalities and the sequence for care; seven of the eleven recommended treatment modalities were considered effective for patients with hip or knee OA by the great majority of the GPs and five of the seven SCS recommendations regarding the sequence for care were consistent with the attitudes of most GPs. However, we found a notably high number of GPs who reported that tramadol, topical NSAIDs, and glucosamine were not effective and who reported that non-recommended modalities were effective in patients with hip or knee OA. Also, many GPs considered an X-ray necessary to diagnose OA and considered NSAIDs the drug of first choice (instead of acetaminophen). GPs’ agreement with SCS recommendations regarding the sequence for care was only weakly associated with a positive attitude towards the effectiveness of recommended modalities, a negative attitude towards the effectiveness of non-recommended modalities, and working in a duo or group practice.

As mentioned above, many GPs reported thought that several types of pain medication that are recommended in the SCS are not effective in patients with hip or knee OA. An explanation for the negative attitude towards tramadol could be the high prevalence of side effects [22,23], which may result to therapy switching or discontinuation, and thus a negative evaluation of its effectiveness by GPs [24,25]. Furthermore, the GPs’ negative attitudes towards the effectiveness of topical NSAIDs, glucosamine, and hyaluronic acid intra-articular injections might be explained by the discrepancies between the recommendations of the SCS and the Dutch NHG-standard for non-traumatic knee complaints in adults [6]. As the SCS was developed through a consensus procedure based on several national and international guidelines, the SCS recommendations differed slightly from those of the Dutch NHG-guideline. For example, the NHG-standard recommends GPs not to use glucosamine in patients with OA and gives no recommendations regarding topical NSAIDs, while the SCS does. In general, NHG-standards have a great impact on GPs’ knowledge [20] and thus could have influenced GPs’ attitudes regarding the effectiveness of these modalities. Another possible explanation for the GPs’ negative attitude towards topical NSAIDs, glucosamine, and hyaluronic acid intra-articular injections is that these are not reimbursed in the Netherlands. The GPs’ positive attitude regarding the non-recommend modalities, like manual therapy and other passive modalities, could be explained by the preferences for these modalities by the patients and physical therapists. This explanation is supported by the fact that these modalities are frequently used in patients with OA [26].

The SCS points out that medical history and physical examination are sufficient to diagnose (symptomatic) hip or knee OA, as radiographic confirmation of OA has little impact on the management, particularly in the early stages of the disease [3,5,27]. Interestingly, many GPs reported that X-ray is necessary to diagnose hip or knee OA. This finding is concordant with other studies that assessed GPs’ behaviour for ordering X-rays in the management of OA or back pain [28-30]. The GPs legitimise their use of radiographs by expressing the view that it aids the discussion of management with the OA patient, is required for specialist referral, and can be used to reduce referrals [29]. Moreover, GPs believe that X-rays provide reassurance to patients which can outweigh the risks; furthermore, denying X-rays could adversely affect the doctor-patient relationship [30]. Although patients who have had X-ray seemed to be more satisfied, they reported more pain, lower overall health status, though no difference in disability, and consult their doctor more frequently [30,31]. In light of this, GPs need to be informed about the limited value of X-rays in early OA.

We found three factors that are associated with the GPs’ attitude regarding the sequence for care. The first two factors, that concern the GPs’ agreement with the effectiveness of recommended and non-recommended treatment modalities, suggest that GPs who are aware of the effectiveness of these modalities agree with the SCS recommendations regarding the sequence for care. This result might be explained by the fact that these recommendations were based on evidence outlined in CPGs. Furthermore, the association between the GPs’ attitude regarding the sequence for care and the type of practice is in concordance with other studies suggesting that the organizational setting of the practice is the most consistent predictor of the GPs’ behaviour and can influence GPs’ performance [32,33]. Generally, the isolated physician in solo practice provides a more limited range of services and show lower levels of clinical competence [33]. Moreover, GPs in solo practices appear to have a more aggressive treatment style than those physicians in group practices, which might be explained by financial incentives, lack of peer influences and availability of colleagues for informal consultation [32].

This study is not without limitations. First of all, only 10% of the variance in GPs’ agreement could be explained by factors related to the GP, the practice, or the management organization for OA patients. We did not examine the contribution of person-related or situational factors, e.g., the GPs’ experience, the patients’ preferences, local infrastructures, and rules or laws on the sequence for care; these factors have been named in literature as potentially able to influence GP’s attitudes [12,16]. Secondly, our study does not cover all professionals because the research aim of this study was restricted to GP’s views and working procedures, while implementation of the multidisciplinary SCS should involve different disciplines. However, this study is part of an umbrella project, the BART-project, which aims to implement the SCS in practice and evaluate the implementation process in one region of the Netherlands, in preparation for the nation-wide implementation. Therefore, the views and working procedures of patients and other health care providers will be studied and described at a later date. Thirdly, the self-designed survey was tailored to our target population and not validated. Fourthly, we measured the GP’s attitudes regarding recommendations of the SCS and not the GP’s actual behaviour. Although a positive relation between attitude and behaviour can be assumed [34] our results do not give insight into the extent that the current clinical practice is concordant with the recommendations of the SCS. Consequently, there might be other barriers that impede a successful implementation. Finally, the response rate (37%), although relatively high for these kind of surveys among GPs, can raise some concerns regarding the validity and generalizability of our findings. Although we did not find large differences in several demographic and practice-related characteristics between the responders and the total population of Dutch GPs, we are aware that non-response bias could have affected the results. It has been stated that “serial” non-responders to GP surveys tent to be older, less likely to possess a postgraduate medical qualification, or belong to a practice that is involved with postgraduate or undergraduate training. [35] As we expect that additional education is associated with a more positive attitude to evidence-based practice, it could be hypothesized that our findings are an overestimation of the degree of agreement with the SCS recommendations.

## Conclusions

Given the above-mentioned findings, the GPs’ attitude regarding recommendations in the SCS is not an insurmountable barrier for implementing the SCS in general practice. GPs are supportive of the recommendations regarding the effectiveness of treatment modalities and the sequence for care. Potential targets for implementation are improving the GPs’ knowledge regarding the effectiveness and optimal sequence for diagnostic procedures and treatment modalities, particularly in GPs who are working in a solo practice. Therefore, we recommend to include these themes in the GP-guideline and embed these in the program of the (post-graduate) training program and/or post-academic training for GPs. We did not identify any barriers that substantially contribute to GPs’ agreement with the SCS recommendations regarding the sequence for care. Further efforts should be taken to identify barriers that could prevent GPs from using the SCS.

## Competing interests

The authors declare that they have no competing interest.

## Authors’ contributions

All authors participated in the design of the manuscript. AJS carried out the data collection and preformed the statistical analysis. AJS and CHM drafted the manuscript. All authors helped to interpret the data and revise the manuscript. Furthermore, all authors read and approved the final manuscript.

## Pre-publication history

The pre-publication history for this paper can be accessed here:

http://www.biomedcentral.com/1471-2296/14/33/prepub
